# Prevalence of Asthma and COPD and Blood Eosinophil Count in a Middle-Aged Belgian Population

**DOI:** 10.3390/jcm8081122

**Published:** 2019-07-28

**Authors:** Sara R. A. Wijnant, Lies Lahousse, Marc L. De Buyzere, Guy G. Brusselle, Ernst R. Rietzschel

**Affiliations:** 1 Department of Respiratory Medicine, Ghent University Hospital, Corneel Heymanslaan 10, 9000 Ghent, Belgium; 2 Department of Bioanalysis, Faculty of Pharmaceutical Sciences, Ghent University, Ottergemsesteenweg 460, 9000 Ghent, Belgium; 3 Department of Cardiology, Ghent University Hospital, and Ghent University, Corneel Heymanslaan 10, 9000 Ghent, Belgium; 4 Department of Respiratory Medicine, Ghent University Hospital, and Ghent University, Corneel Heymanslaan 10, 9000 Ghent, Belgium; 5 Department of Cardiology and Biobanking and Cardiovascular Epidemiology, Ghent University Hospital and Ghent University, Corneel Heymanslaan 10, 9000 Ghent, Belgium

**Keywords:** asthma, chronic obstructive pulmonary disease, eosinophils, middle aged, prevalence, epidemiology

## Abstract

Various phenotypes exist in asthma and Chronic Obstructive Pulmonary Disease (COPD). These are important to identify in order to guide treatment decisions. We aim to investigate the prevalence and clinical characteristics of obstructive airway diseases in the middle-aged population. We estimated the prevalence of COPD and/or asthma in the Asklepios cohort study (Belgium), using information from the third European Community Respiratory Health Survey (ECRHS3), medical records, and spirometry. Respiratory symptoms, respiratory medication, and current disease status distinguished clinical from sub-clinical cases. In addition, we compared the blood eosinophil count/µL (median [IQR]) between cases and controls. Of the 2221 participants (mean age 56.1 ± 5.9 years; 48.7% males), 138 (6.2%) participants had clinical current asthma, 22 (1.0%) participants had sub-clinical ever asthma, 102 (4.6%) had sub-clinical spirometry-defined COPD, 104 (4.6%) participants had clinical spirometry-confirmed COPD, and 11 (0.5%) had asthma and COPD overlap (ACO). Clinical current asthma (160.0 [110.0–250.0]), sub-clinical ever asthma (170.0 [110.0–230.0]), and clinical COPD (160.0 [110.0–220.0])—but less sub-clinical COPD (140.0 [90.0–210.0])—had higher eosinophil counts, compared to controls (130.0 [80.0–200.0]). We conclude that obstructive airway diseases are prevalent in the middle-aged Asklepios cohort. Moreover, the systemic eosinophil count is increased in clinical COPD cases, and in asthma cases regardless of clinical remission.

## 1. Introduction

Obstructive pulmonary diseases have been widely studied, with over 300 million people worldwide affected by both asthma and Chronic Obstructive Pulmonary Disease (COPD) [1,2,3,4,5,6]. Given their increasing burden and financial impact [7,8,9], more attention is rising for the late-onset asthma phenotype [10], that differs from early-onset asthma with respect to genetic susceptibility, environmental exposures, pathogenic mechanisms, comorbidities, prognosis and treatment response [10,11,12,13,14]. Evidence is emerging that, besides tobacco smoking, risk factors, such as respiratory infections, poor nutritional status, chronic asthma, impaired lung growth, poor socio-economic status and genetic factors are also important for COPD development [1]. Moreover, despite the multitude of treatments and interventions available to patients with chronic respiratory diseases, many patients remain refractory, while those who do respond, show marked treatment response variability [15]. Various phenotypes exist in both asthma and COPD, and these are important to recognize, in order to guide treatment decisions.

In asthma, type 2 helper T-cell (Th2)-mediated eosinophilic airway inflammation is typical, whereas in COPD, type 1 helper T-cell (Th1)-mediated neutrophilic inflammation predominates. In each condition, however, a spectrum of phenotypes exists [15]. Blood eosinophil count has been associated with a COPD phenotype, that is at higher risk for frequent exacerbations, but with better corticoid responsiveness [16]. Elevated blood eosinophil count is a marker of active asthma [17,18,19,20,21,22,23,24], and few studies, with clinically remitted asthma subjects, showed an association between persistent eosinophilia and persistent asymptomatic airway hyper-responsiveness (AHR) [25,26,27]. Given that persistent AHR could be a risk factors for relapse [28], the measurement of persistent eosinophilic inflammation, as a proxy of persistent AHR in a more representative sample of the general population, is warranted. Therefore, the objective of this study is to investigate the prevalence and clinical characteristics of obstructive airway diseases in middle-aged participants of the Asklepios Study, a community-based cohort study in Belgium.

## 2. Experimental Section

Subjects were chosen from the Asklepios Study, a random sample of participants recruited from the general population with the approval of the ethics committee of Ghent University Hospital, and in accordance with the Declaration of Helsinki (see Appendix A for a more detailed description) [29]. This study uses measurements from round 2 of the study (2011–2016) as no spirometry was performed in round 1 (2002–2004). All participants gave written informed consent.

A single trained examinator performed spirometry in all subjects according to the American Thoracic Society/European Respiratory Society (ATS/ERS) guidelines, using a portable spirometer (Spirobank G; MIR; Rome, Italy). Forced Expiratory Volume in 1 s (FEV1), Forced Vital Capacity (FVC), FEV1/FVC, the spirogram (volume-time curve) and maximal expiratory flow-volume curves were measured. Spirometry results that did not meet the ATS/ERS criteria for acceptability and reproducibility were classified as non-interpretable. Percent predicted of FEV1 and FVC were calculated using GLI reference equations [30]. The interpretation of all spirometry results was carried out by an experienced researcher (LL), and in case of disagreement with the computerized interpretation, using the assistance of a respiratory physician (GB).

Case finding by three researchers (SW, LL and ER) bundled all the participants with either, respiratory symptoms (question 1–9 from the third European Community Respiratory Health Survey ECRHS3 question 1–9; see Appendix B), a history of asthma or COPD (general practitioner’s [GP] medical file or ECRHS3), respiratory medication usage (GP medical file or ECRHS3), or an obstructive pulmonary function test (FEV1/FVC < 0.7). We relabeled 24 cases—that were labelled by the GP as COPD—as asthma because their FEV1/FVC ratio was 0.7 or higher (mean FEV1/FVC 0.77 ± 0.42).

Clinical asthma cases were distinguished from sub-clinical asthma cases when participants with asthma (a history of asthma in the GP medical file or ECRHS3 question 12–13) either a. used respiratory medication (GP medical file, or ECRHS3 question 10 or question 19), or b. reported any respiratory symptoms during the past 12 months (ECRHS3 question 1–9), or c. self-reported current asthma (ECRHS3 question 13). Clinical COPD cases were distinguished from sub-clinical COPD cases when participants with COPD (FEV1/FVC < 0.7) either a. used respiratory medication (GP medical file or ECRHS3 question 10 or question 19), or b. reported any respiratory symptoms during the past 12 months (ECRHS3 question 1–9), or c. self-reported known COPD (ECRHS3 question 18). Clinical COPD cases were divided into mild (FEV1 ≥ 80%) and moderate to severe COPD (FEV1 < 80%). Asthma and COPD overlap (ACO) cases had a diagnosis of both asthma and COPD (GP medical file or ECRHS3) in the presence of an obstructive pulmonary function (FEV1/FVC < 0.7), and were not included in asthma and COPD groups for analyses.

The normal group (no history of asthma or COPD, nor respiratory symptoms or respiratory medication use and normal spirometry [FEV1/FVC ≥ 0.7 and FVC ≥ 80%]) and the subnormal group (respiratory symptoms or medication use but no diagnosis of asthma or COPD and normal spirometry) were pooled to form the control group. We excluded cases with a spirometry suggestive of restrictive pulmonary disease (FVC < 80% or based on an abnormal flow-volume curve; *n* = 15). Subjects with Preserved Ratio Impaired Spirometry (PRISm; and FEV1/FVC ≥ 0.7 and FEV1 < 80%; *n* = 63) were not excluded.

Respiratory medication (R03) was defined according to The Anatomic Therapeutic Chemical Classification (ATC) System. Blood eosinophil count was measured on EDTA whole blood using an ISO 17,025 Beltest accreditation in a reference laboratory (Laboratorium Klinische Biologie, UZ Ghent; ISO 9002; NBN EN 45001). We compared smoking history (pack-years as years times daily cigarettes divided by 20), arterial hypertension (systolic blood pressure ≥140mmHg, diastolic blood pressure ≥90 mmHg or use of anti-hypertensive medication), body composition (overweight: Body Mass Index [BMI] ≥25 kg/m^2^; obesitas: BMI ≥30 kg/m^2^), type 2 diabetes (fasting blood glucose ≥126 mg/dL or use of blood glucose lowering medication), renal function impairment (mild: estimated glomerular filtration rate [eGFR, using the CKD-EPI equations ] <90 mL/min/1.73 m^2^; moderate to severe: eGFR <60 mL/min/1.73 m^2^), and atopy (self-reported allergic rhinitis or hay fever [ECRHS3 question 11] or use of anti-allergic medications [ATC R06]) between cases and controls. 

Prevalence was calculated by dividing the total number of cases by the total number of participants. Data are expressed as mean ± standard deviation (SD) for normally distributed variables or as median (interquartile range [IQR]) for non-normally distributed variables. We compared basic characteristics between groups with the Student’s *t*-test for continuous parametrical variables, the Mann-Whitney-U test for continuous non-parametrical variables, or the Chi-square/Fisher exact test for categorical variables. Odds ratios (OR) from logistic regression analyses were adjusted for age, sex, BMI, pack-years and eosinophil percentage. Possible confounders were chosen based on univariate testing and literature data. All statistical analyses were carried out using SPSS version 25 (IBM, New York, NY, USA) and R version 3.5.1 (R Project for Statistical computing, Vienna). A *p*-value < 0.05 was considered statistically significant. The data supporting the findings of this study are only available from the principal investigator of the Asklepios Study upon reasonable request and after positive evaluation by the Asklepios Steering Committee. 

## 3. Results

**Figure 1:**
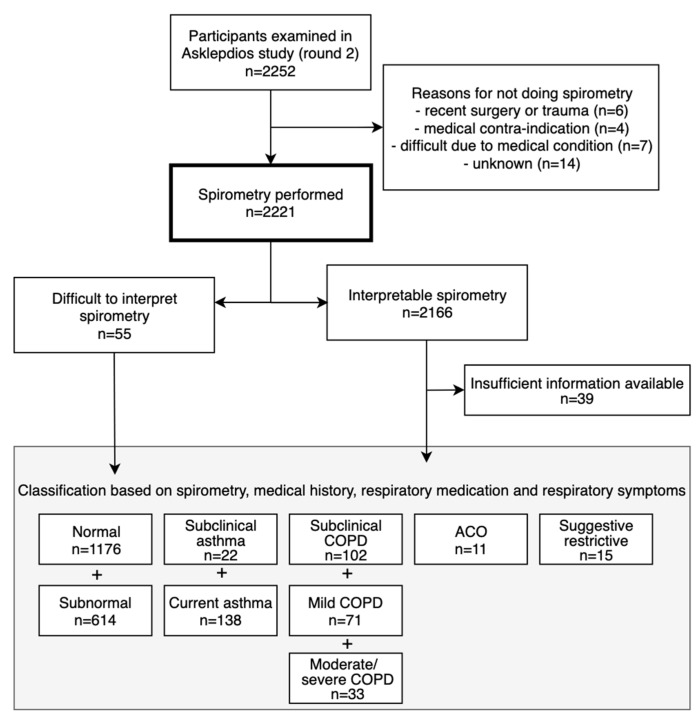
Flowchart of participants.

**Figure 2:**
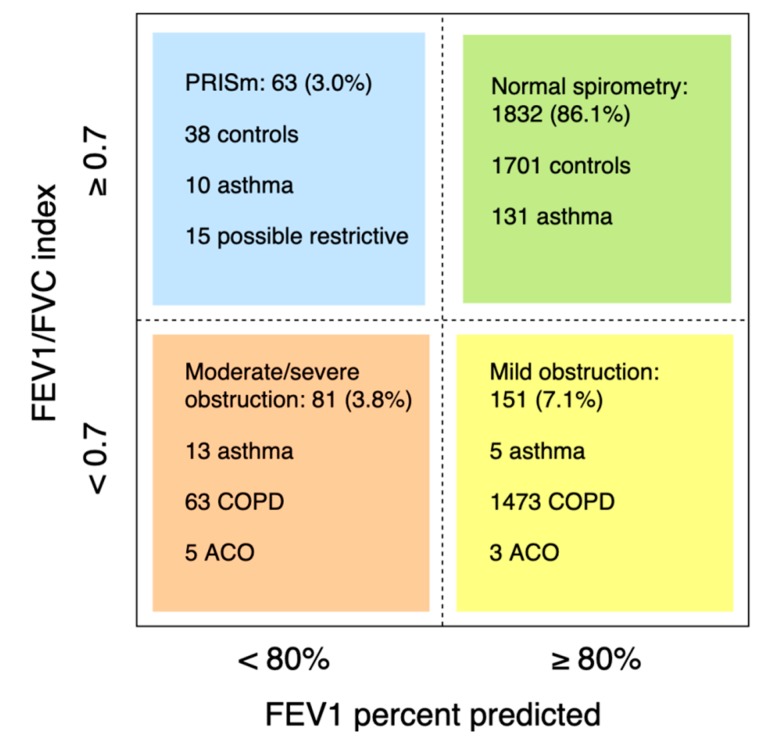
Lung function of subjects with an interpretable spirometry test. Participants with insufficient clinical information, available to categorize in a lung function group, were excluded (*n* = 39).

Of 2221 participants with lung function measurements (mean age 56.1 ± 5.9 years, 48.7% males), 377 had asthma and/or COPD (Figure 1). Of 160 asthma cases (prevalence 7.2%; 6.8% in males and 7.5% in females), 138 had current asthma (45.7% male) and 22 had sub-clinical asthma (50.0% male). Another eleven cases with asthma had concomitant COPD (i.e. ACO; prevalence 0.5%, 54.5% male). Of 206 COPD cases (prevalence 9.3%; 12.0% in males and 6.7% in females, *p* < 0.001), 104 were clinical cases: 62 with mild COPD (69.4% male) and 42 with moderate to severe COPD (61.9% male). 102 subjects had sub-clinical COPD (59.8% male). The prevalence of obstructive spirometry, according to the LLN method, was 6.5%. Of 2166 subjects with interpretable spirometry measures, 63 had PRISm (mean age 56.4 ± 6.2 years, 44.4% male; Figure 2).

**Figure 3:**
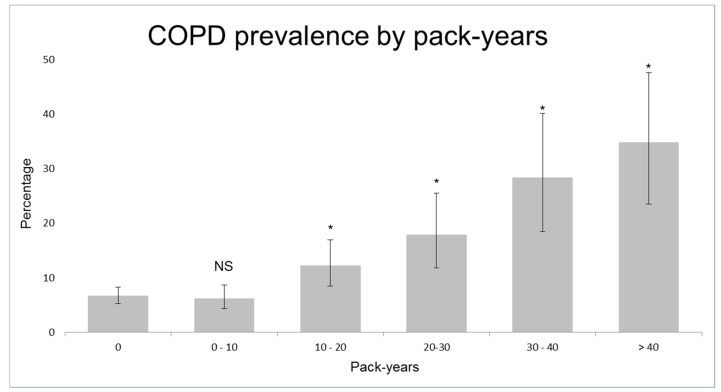
Prevalence of Chronic Obstructive Pulmonary Disease (COPD) according to pack-years. NS = not significant; * = *p* < 0.05 compared to subjects that have never smoked.

Table 1 shows the characteristics of controls and cases with asthma and COPD. Smokers showed an increased risk of COPD, starting from 10 pack-years (adjusted OR = 1.8, 95% CI 1.1–2.8; Figure 3, Figure S1). 40.4% (*n* = 42) of clinical COPD cases had smoked 10 pack-years or less. COPD subjects that had smoked 10 pack-years or less were younger (mean age 56.0 ± 6.0 years versus 58.7 ± 4.9, *p* = 0.001) and had more often atopy (24.7% versus 12.1%, *p* = 0.026; see Table S1 for other characteristics) than other COPD subjects. Current smokers had a higher risk of asthma compared to never/ex-smokers (adjusted OR = 1.5, 95% CI 0.9–2.6). Of the symptomatic COPD cases, only 31.3% (30/96) were previously diagnosed with COPD and/or received respiratory treatment, in contrast to 99.1% (116/117) of symptomatic asthma cases (Figure S2).

Within 2192 participants (98.7%) with available blood eosinophil counts, the median absolute blood eosinophil count was higher in current asthma, ever asthma, clinical COPD and ACO subjects compared to controls, but less in sub-clinical COPD subjects (Table 2). In logistic regression analysis adjusted for age, sex, BMI, current smoking, type 2 diabetes and corticosteroid intake, eosinophil percentage was associated with current asthma (OR = 2.4, 95%CI 1.5–3.9), ever asthma (OR = 2.9, 95%CI 1.1–7.3) and clinical COPD (OR = 1.7 95%CI 1.1–2.7), but not with sub-clinical COPD (OR = 1.2, 95%CI 0.7–1.9). The association between eosinophil percentage and ever asthma lost statistical significance when adjusted for atopy (OR = 2.4, 95%CI 0.9–6.2).

## 4. Discussion

In this cohort of middle-aged individuals in Belgium (mean age 56 years), the prevalence of asthma is 7.2%, the prevalence of COPD is 9.3%, and the prevalence of ACO is 0.5%. We found an increased systemic eosinophilia in asthma cases, regardless of clinical remission, and in clinical—but not sub-clinical—COPD.

The prevalence estimates of asthma and COPD, in this study, are in line with previous findings. The literature on the epidemiology of asthma in middle-aged and older subjects is rising and their prevalence has been estimated between 5% and 14% [31]. The prevalence of COPD ranged between 2.6–26.1% in European adult populations over 40 years [4,5], yet COPD was often under-diagnosed and under-treated in this study, despite that these cases often reported respiratory symptoms. Therefore, more awareness of lung-function deterioration in middle-aged populations, and the adherence to clinical practice guidelines, is needed.

Interestingly, we showed that the systemic eosinophil count is increased in asthma cases, despite clinical remission. Increased eosinophilic inflammation in asthma subjects, after clinical remission, has been observed [25,27,32,33,34,35], but prior studies consisted of small sample sizes and/or were conducted in hospital settings. This study of a more representative sample of the general population reinforced these preliminary findings. Although promising, further prospective research is needed to confirm whether the persistent increase of subjects’ blood eosinophil count—as a proxy of persistent AHR—is also a predictor of asthma relapse. If so, it could potentially serve as a clinical decision directory for longer lasting maintenance therapy.

Clinical–but not sub-clinical–COPD was characterized by increased eosinophilic inflammation in this study. Only few studies researching COPD and blood eosinophilia, in a general population setting, were previously done [36,37,38]. The majority (70.7%) of COPD cases in a US population exhibited eosinophil percentages of >2% [37]. Danish COPD patients, with a baseline eosinophil count of >340 cells/µL, had a higher increased risk of severe exacerbations [36]. COPD patients from a UK study with eosinophil counts of ≥450 cells/µL during stable disease had a higher exacerbation rate during the following year [38]. Given the high intra-person variability of blood eosinophil count, multiple measures of systemic eosinophilia could possibly improve the accuracy of these predictions, which remains a challenge for future studies [39].

Despite the strong association between cigarette smoking and COPD, we found that a significant part of COPD subjects had a smoking history of less than 10 pack-years. The incidence of COPD in never-smokers indicates that other factors, such as genetic susceptibility, impaired lung growth, respiratory infections, and environmental exposures, including occupational exposures and (outdoor and indoor) air pollution, likely contribute to the development of COPD. Consistent with this study, three other population-based studies reported similar results: 16.3% of COPD subjects in the Netherlands had never smoked [40], 42.9% of COPD subjects in European countries had smoked less than 20 pack-years [41], and more than half (59.6%) of COPD subjects from a Korean population had smoked less than 100 cigarettes in their entire life [42]. Nevertheless, most randomized clinical trials, that examine the efficacy and safety of pharmacologic treatments for COPD, only include COPD patients with a history of cigarette smoking of at least 10 pack-years. However, treatment effects could differ in COPD subjects with different disease mechanisms.

This study has some limitations. First, the use of pre-bronchodilator spirometry does not enable distinctions to be drawn between asthma cases with current airflow obstruction and COPD or ACO. However, we carefully checked the subjects’ medical records, and none of those classified as having COPD (based on presence of pulmonary obstruction) had a history of asthma. Nevertheless, misclassification remains possible when, for example, a diagnosis of asthma was wrongly missed by a GP. The relatively frequent occurrence of atopy, among COPD subjects with a smoking history of 10 pack-years or less, indicates the presence of such misclassification to some extent. Second, lung functions were measured at a single time-point using a fixed cut-off for FEV1/FVC; thus, there may have been some misclassification of obstructive pulmonary function, according to the current guidelines [43]. Third, 17 participants were ineligible to perform spirometry due to bad health (e.g., recent surgery, esophageal varices, difficult exhalation, nervousness), which could have resulted in under-representation of subjects with more severe asthma or COPD.

The main strengths of this study are its large sample size (*n* = 2221) and population-based nature. The application of exclusion criteria at baseline is an acknowledged risk for selection bias, and is likely to under-estimate the prevalence of COPD, since both COPD and cardiovascular diseases share a common ground, namely cigarette exposure. However, we believe that the results from this study are generalizable to other populations, given the small number of participants excluded, and given the similarities between this population and others (e.g. prevalence of asthma and COPD, and population characteristics). Adding to this, the loss of follow-up between round 1 and round 2 of the Asklepios study was minimal, with re-examination in 91% of individuals. The second strength is that all measurements were carried out in a standardized manner by the same investigator. Third, COPD diagnosis was strictly validated based on spirometry, and the asthma cases were validated using data from the subjects’ medical files and questionnaire data. The completeness of the participants’ medical information assumes that few diagnoses were missed. Fourth, the prevalence of asthma and COPD in Belgium has not been previously reported. Last, we distinguished clinical from sub-clinical asthma cases, thereby providing useful information on the characteristics of asthma subjects after clinical remission.

## 5. Conclusions

In summary, this study showed that, both asthma and COPD, are common diseases in the middle-aged population of the Asklepios study in Belgium, and that COPD is often under-diagnosed and under-treated, despite respiratory symptomatology. A greater awareness of lung-function deterioration in the middle-aged population, and adherence to clinical practice guidelines is needed. Interestingly, the systemic eosinophil count was increased in asthma cases, despite clinical remission. Future studies should focus on the utility of systemic eosinophil count to predict asthma relapse after clinical remission.

## Figures and Tables

**Table 1:** Baseline characteristics of subjects in the Asklepios Study.

Subject Characteristics	Control Subjects (*n* = 1790)	Asthma (*n* = 160)	COPD (*n* = 206)
Age (year)	56.0 ± 5.9	55.2 ± 6.2	57.3 ± 5.7 *
Male (%)	841 (47.0%)	74 (46.3%)	130 (63.1%) *
Higher education	685 (38.5%)	55 (34.6%)	79 (38.7%)
Pack-years nicotine (year)	0.0 (0.0–7.5)	0.0 (0.0–6.5)	8.8 (0.0–27.0) *
Former smoker	650 (36.3%)	60 (37.2%)	71 (34.5%)
Current smoker	152 (8.5%)	19 (11.9%)	59 (28.6%) *
Cardiovascular risk factors			
Systolic blood pressure (mmHg)	130.2 ± 15.1	129.7 ± 13.3	129.4 ± 14.9
Diastolic blood pressure (mmHg)	81.6 ± 9.9	82.1 ± 9.6	80.2 ± 10.1 *
BMI (kg/m^2^)	26.8 ± 4.5	27.9 ± 5.4 *	25.8 ± 3.9 *
Total cholesterol (mg/dL)	209.7 ± 40.0	209.3 ± 36.6	204.9 ± 38.1
Glycaemia (mg/dL)	96.6 ± 16.3	97.2 ± 16.2	96.4 ± 13.7
Inflammation			
High sensitive CRP (mg/L)	0.99 (0.53–2.16)	1.28 (0.67–2.66) *	0.91 (0.48–1.85)
White blood cell count (10^9^/L)	6.7 ± 1.8	6.8 ± 1.8	7.1 ± 2.0 *
Eosinophil percentage (%)	2.0 (1.3–3.0)	2.4 (1.7–3.8) *	2.3 (1.5–3.4) *
Lung function			
FEV1 percent predicted (%)	104.6 ± 12.9	96.4 ± 15.9 *	87.3 ± 18.2 *
FVC percent predicted (%)	104.7 ±12.9	100.4 ±13.8 *	105.6 ± 18.7 *
FEV1/FVC (%)	78.9 ± 4.2	75.8 ± 6.3 *	64.5 ± 5.9 *
Respiratory symptoms in the past 12 months	603 (34.4%)	117 (73.6%) *	96 (48.0%) *
Comorbidities			
Arterial hypertension	781 (43.6%)	78 (48.8%)	95 (45.9%)
Overweight (BMI ≥ 25 and < 30 kg/m^2^)	759 (42.4%)	72 (45.0%)	85 (41.1%)
Obese (BMI ≥ 30 kg/m^2^)	379 (21.2%)	42 (26.3%)	23 (11.2%) *
Type 2 diabetes	118 (6.6%)	14 (8.8%)	15 (7.3%)
Mild renal function impairment (eGFR 60–89)	1137 (63.6%)	98 (61.3%)	132 (63.6%)
Moderate to severe renal function impairment (eGFR < 60)	70 (3.9%)	9 (5.6%)	8 (3.9%)
Atopy	278 (16.7%)	81 (53.6%) *	35 (18.6%)
Laboratory parameters			
eGFR (mL/min/1.73 m^2^)	84.9 ± 17.1	83.5 ± 16.8	84.9 ± 16.4
Creatinine (mg/dL)	8.8 ± 1.7	8.9 ± 1.8	9.1 ± 1.7 *
Microalbuminuria (mg/L)	6.8 (4.4–11.6)	6.3 (4.7–10.9)	6.4 (4.2–10.3)
Hematocrit (%)	41.5 ± 3.3	41.7 ± 3.3	42.2 ± 3.0 *
Thrombocytes (10^9^/L)	242.9 ± 56.3	249.5 ± 52.6	246.0 ± 50.5
Respiratory medication			
Respiratory medication use	49 (2.7%)	100 (62.5%) *	30 (14.6%) *
Self-reported respiratory medication use	40 (2.3%)	95 (59.7%) *	29 (14.5%) *
GP reported respiratory medication use (ATC R03): *SABA* *LABA* *LAMA* *SAMA* *ICS* *Leukotriene-receptor antagonist*	19 (1.1%): *1 (0.1%)* *10 (0.6%)* *0 (0.0%)* *0 (0.0%)* *16 (0.9%)* *2 (0.1%)*	80 (50.0%) *: 11 (6.9%) * 60 (37.5%) * 0 (0.0%) *1 (0.6%)* 69 (43.1%) * 20 (12.5%) *	19 (9.2%) *: *0 (0.0%)* 17 (8.3%) * 2 (1.0%) * 0 (0.0%) 16 (7.8%) * 0 (0.0%)
Other medication			
Anti-histaminica (ATC R06)	49 (2.7%)	27 (16.9%) *	4 (1.9%)
OCS (ATC H02)	16 (0.9%)	1 (0.6%)	3 (1.5%)

eGFR = estimated Glomerular Filtration Rate; SABA = short-acting beta-agonist; LABA = long-acting beta-agonist; LAMA = long-acting muscarinic antagonist; ICS = inhalation corticosteroids; OCS = oral corticosteroids; * *p* < 0.05 compared to control subjects.

**Table 2:** Eosinophilic inflammation in subjects in the Asklepios Study.

	Controls	Current Asthma	Ever Asthma	Sub-Clinical COPD	Clinical COPD	ACO
Eosinophil count (cells/µL)	130.0 (80.0–200.0)	160.0 (110.0–250.0) *	170.0 (110.0–230.0)	140.0 (90.0–210.0) **	160.0 (110.0–220.0) *	180.0 (80.0–340.0)
Eosinophil count ULN/≥ 310 (%)	159 (9.0%)	23 (17.2%) *	5 (22.7%) *	11 (10.8%)	15 (14.7%)	3 (27.3%)
Eosinophil percentage (%)	2.0 (1.3–3.0)	2.4 (1.6–3.8) *	2.3 (1.8–5.4)	2.2 (1.4–3.3)	2.3 (1.6–3.5) *	2.4 (1.3–5.0)
Eosinophil percentage ULN/≥4.6, (%)	165 (9.3%)	22 (16.4%) *	6 (27.3%) *	9 (8.8%)	19 (18.6%) *	3 (27.3%) *

Comparison of median blood eosinophil count in cases versus controls; ACO = Asthma and COPD overlap; ULN = upper limit of normal (90th percentile). * *p* < 0.05. ** *p* < 0.1.

## Supplementary Materials

The following are available online at https://www.mdpi.com/2077-0383/8/8/1122/s1, Figure S1: Estimated probability of COPD according to pack-years, Figure S2: Characteristics of cases with current asthma and clinical COPD, Table S1: Characteristics of COPD cases stratified by >10 pack-years and ≤10 pack-years of smoking history.

## Appendix A. Study Population and Baseline Data Collection

The Asklepios study comprises 2524 subjects (1301 women) approximately 35 to 55 years of age at the baseline examination in 2002–2004 (round 1), with a complete re-examination of 91% of subjects after a median of 10.1 (9.8–10.8) years of follow-up (round 2: in 2011–2016). At the baseline investigation, subjects were apparently healthy subjects free from overt cardiovascular disease. The subjects were randomly sampled from two Belgian communities (Erpe-Mere and Nieuwerkerken) with approval of the ethics committee of Ghent University Hospital. These communities have a very active and organized participation of a group of 89 general practitioners (regional coverage for Erpe-Mere and Nieuwerkerken >95%). 8104 of the approximately 25,000 inhabitants were within the target age group (data provided by local authorities) and were randomly sampled from the population list through post mailing. Partners and relatives of those recruited by mailing were also eligible for study entry (if they inhabited Erpe-Mere or Nieuwerkerken) and a total of 703 subjects were recruited in this manner. Subjects were initially screened by their general practitioner (GP), who reviewed inclusion and exclusion criteria and provided a detailed personal medical history, conventional cardiovascular risk profile and an overview of drug use in the preceding 6 months before inclusion. Exclusion criteria at baseline were: 1. clinical presence of atherosclerosis/atherothrombosis; 2. major concomitant illness; 3. Diabetes mellitus (DM) type 1, and type 2 if proven macro-vasculopathy or significant renal impairment; 4. conditions precluding accurate haemodynamic assessment (atrial fibrillation, pregnancy); 5. inability to provide informed consent. Participants completed a study questionnaire, which was reviewed by the study nurse together with the subject during the participant examination. Measurements of round 2 [29] were performed during a continuous period, between 2011 and 2016 at the same single study site in Erpe–Mere and essentially mirrored all examinations performed in round 1 (informed consent, study questionnaire, basic clinical data, extensive blood sampling, echocardiographic examination, and vascular echographic and arterial tonometric measurements), with the addition of spirometry testing. In this study, we used measurements of round 2 (as no spirometry was performed in round 1).

## Appendix B. Dutch Translation of the Third European Community Respiratory Health Survey

1. Hebt u op enig ogenblik in de laatste 12 maanden piepen of fluiten in uw borstkas gehad?
  - Was u kortademig wanneer dit piepend geluid aanwezig was?
  - Hebt u dit piepen of fluiten gehad wanneer u niet verkouden was?
2. Bent u op enig ogenblik in de laatste 12 maanden wakker geworden met een gevoel van beklemming in de borstkas?
3. Bent u op enig ogenblik in de laatste 12 maanden wakker geworden door een aanval van kortademigheid?
4. Heeft u op enig ogenblik in de laatste 12 maanden een aanval van kortademigheid gehad die begon na inspanning?
5. Heeft u op enig ogenblik in de laatste 12 maanden een aanval van kortademigheid gehad die overdag begon wanneer u in rust was?
6. Bent u op enig ogenblik in de laatste 12 maanden wakker geworden door een hoestbui?
7. Hoest u bijna dagelijks gedurende tenminste 3 maanden per jaar?
8. Hoest u bijna dagelijks gedurende tenminste 3 maanden per jaar fluimen o pvanuit uw borstkas?
9. Hebt u in de laatste 12 maanden een aanval van astma gehad?
10. Neemt u momenteel geneesmiddelen (bv. puffers, aerosols, tabletten) voor astma?
11. Hebt u enige vorm van neusallergie, “hooikoorts” inbegrepen?
  - Hoe oud was u toen u voor het eerst hoorkoort of neusallergie had?
12. Hebt u tot heden ooit astma gehad?
  - Werd dit door een arts bevestigd?
  - Hoe oud was u toen u voor het eerste een astma-aanval had?
13. Hebt u momenteel astma?
14. Has uw vader, moeder of één van uw broers of zusters ooit astma?
15. Rookte uw moeder ooit regelmatig tijdens uw kinderjaren of vooraleer u geboren was?
16. Rookte uw moeder wanneer ze van u zwanger was?
17. Hebt u voor de leeftijd van 5 jaar een ernstige long- of luchtweginfectie gehad?
18. Heeft een arts tegen u ooit gezegd dat u chronische bronchitis, COPD of emfyseem heeft?
  - Hoe oud was u toen een arts gezegd heeft dat u chronische bronchitis, COPD of emfyseem had?
19. Heeft u in de laatste 12 maanden regelmatig (d.w.z. op de meeste dagen) een corticosteroiden-inhalator (bv. pulmicort, becotide) gebruikt?
20. Heeft u ooit gedurende een jaar gerookt?
  - Hoe oud was u toen u startte met roken?
  - Heeft u gedurende de laatste maand gerookt?
  - Hoe oud was u toen u voor het laatst rookte? (indien u nog steeds rookt, vul dan uw huidige leeftijd in)
21. Hoeveel jaar woont u reeds in uw huidige woning? Indien minder dan 12 maanden, vul dan 1 in.
