# Supplementary material for: Prevalence of Asthma and COPD and Blood Eosinophil Count in a Middle-Aged Belgian Population
Source: J Clin Med. 2019 Jul 28;8(8):1122. doi: 10.3390/jcm8081122 (PMC6723474; doi:10.3390/jcm8081122)
Supplement: Supplementary file 1 [file jcm-08-01122-s001.pdf]

Supplementary Information template:

SUPPLEMENTARY INFORMATION

Figure S1. Estimated probability of COPD according to pack-years

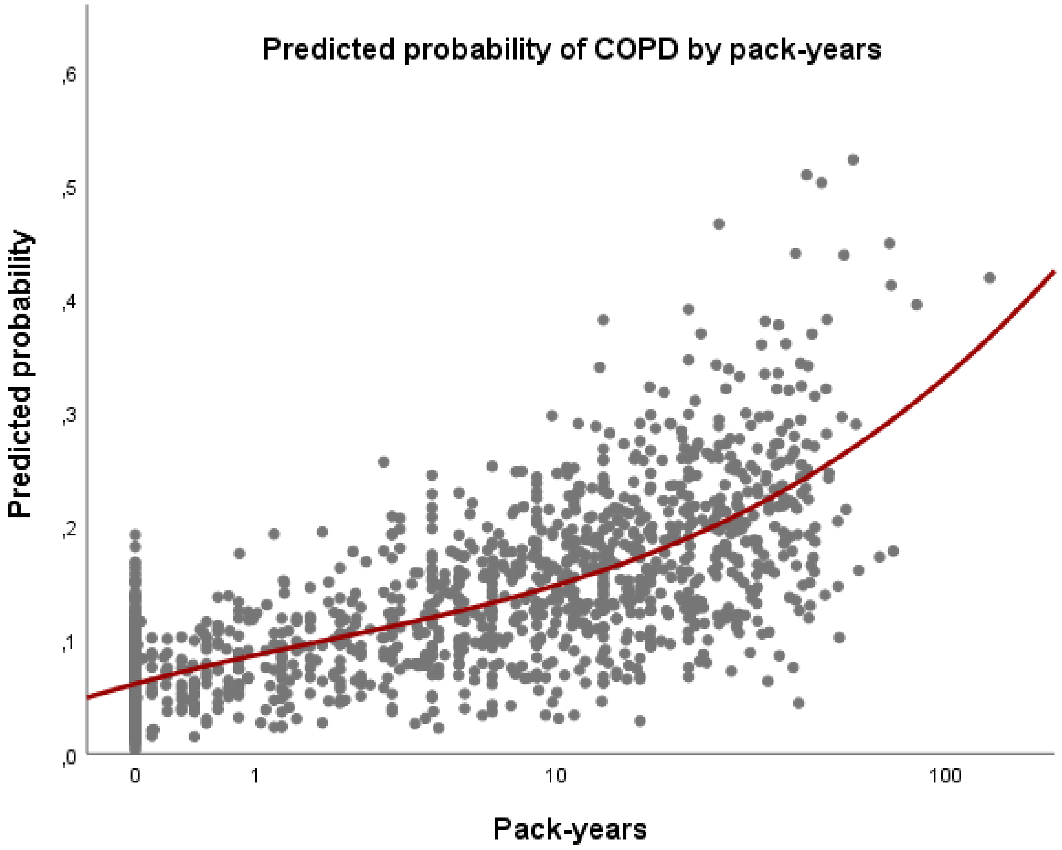

Estimated probability of COPD (y-scale) according to pack-years (x-scale) after adjustment for age, sex, BMI and eosinophil percentage.

Figure S2. Characteristics of cases with current asthma and clinical COPD

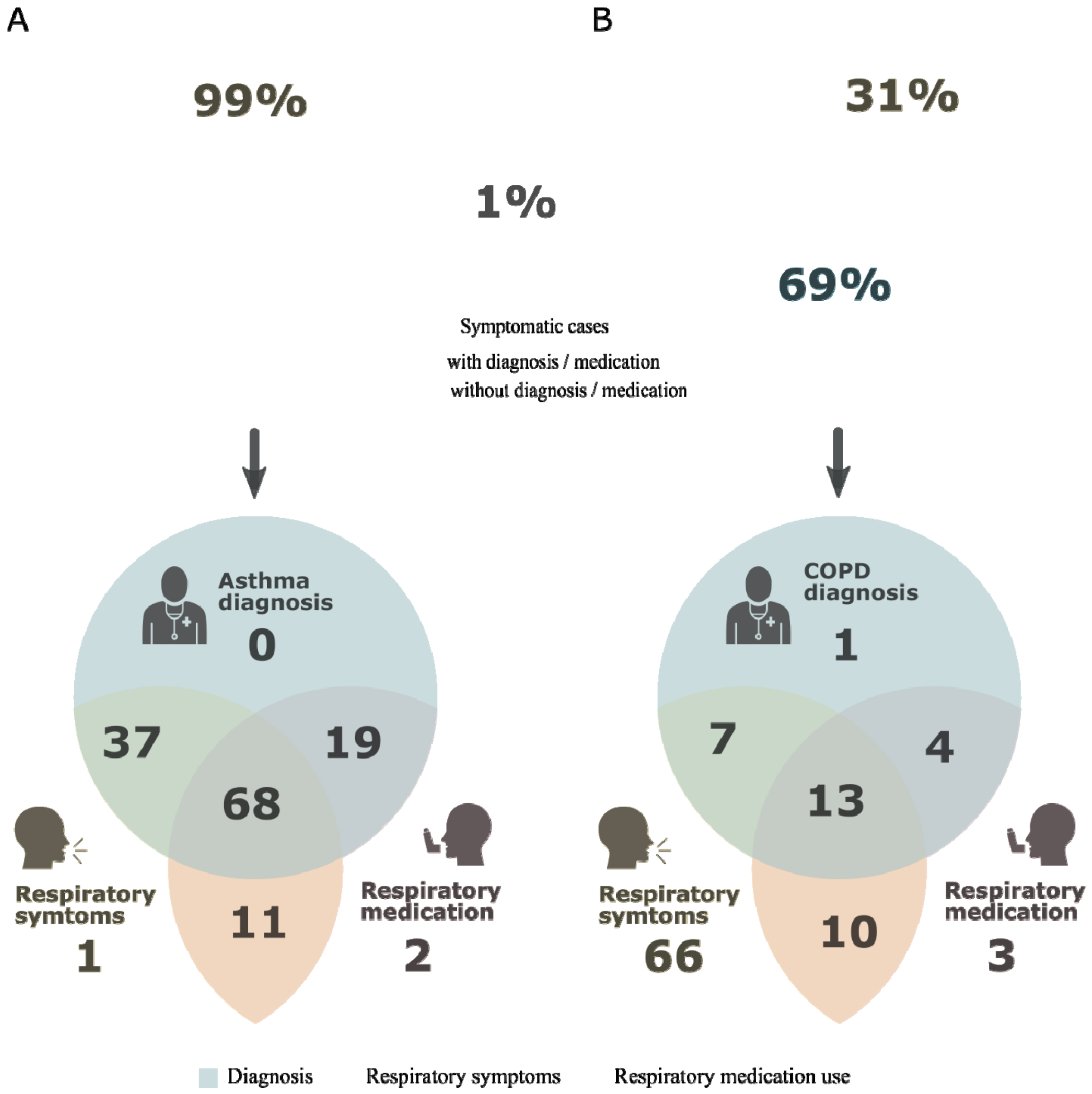

Proportion of symptomatic cases with or without diagnosis and / or respiratory medication use in current asthma (A) and clinical COPD (B). In both current asthma and clinical COPD, 1 subject did not fill in the ECRHS3 questionnaire and they were classified in the figure as if they did not have respiratory symptoms.

Manuscript submission template:

Table S1. Characteristics of COPD cases stratified by >10 pack-years and ≤10 pack-years of smoking history

**Subject characteristics**	**COPD ≤10 pack-years (n=107)**	**COPD >10 pack-years (n=99)**	**P**
Age (year)	56.0 ± 6.0	58.7 ± 4.9	0.001
Male (%)	62 (57.9%)	68 (68.7%)	0.110
Higher education	44 (41.1%)	35 (36.1%)	0.461
Clinical COPD	42 (39.3%)	62 (62.6%)	
**Lung function**			
Current respiratory symptoms (%)	36 (33.6%)	60 (60.6%)	<0.001
FEV1 percent predicted (%)	93.1 ± 15.1	81.0 ±19.2	<0.001
FVC percent predicted (%)	111.3 ± 17.6	99.4 ± 18.0	<0.001
FEV1/FVC (%)	65.8 ± 4.5	63.1 ± 6.9	0.001
**Characteristics of metabolic syndrome**			
BMI (kg/m²)	25.7 ± 3.7	25.8 ± 4.2	0.856
Systolic blood pressure (mmHg)	129.7 ± 13.2	129.0 ± 16.6	0.720
Diastolic blood pressure (mmHg)	81.7 ± 9.4	78.5 ± 10.7	0.024
Total cholesterol (mg/dL)	210.5 ± 33.0	198.8 ± 42.3	0.026
Triglycerides (mg/dL)	81.0 (60.0-118.0)	93.5 (69.0-127.3)	0.058
Glycaemia (mg/dL)	93.9 ± 10.4	99.3 ± 16.2	0.005
**Inflammatory parameters**			
High sensitive CRP (mg/L)	0.85 (0.46-1.83)	1.03 (0.54-2.03)	0.289
White blood cell count (10^9/L)	6.7 ± 1.8	7.5 ± 2.1	0.002
Eosinophils percentage (%)	2.2 (1.3-3.4)	2.3 (1.6-3.4)	0.450
**Comorbidities**			
Arterial hypertension	46 (43.0%)	49 (49.5%)	0.349
Overweight (BMI≥25 and<30 kg/m²)	45 (42.1%)	40 (40.4%)	0.810
Obese (BMI≥30 kg/m²)	11 (10.3%)	12 (12.1%)	0.675
Type 2 diabetes	4 (3.7%)	11 (11.1%)	0.042
Mild renal function impairment (eGFR 60-89)	73 (68.2%)	58 (58.6%)	0.151
Moderate to severe renal function impairment (eGFR<60)	5 (4.7%)	3 (3.0%)	0.542
Atopy	24 (24.7%)	11 (12.1%)	0.026
**Laboratory parameters**			
eGFR (ml/min/1.73m2)	84.2 ± 17.0	85.7 ± 15.8	0.523
Creatinine (mg/dL)	9.1 ± 1.7	9.1 ± 1.8	0.834
Microalbuminuria (mg/L)	6.1 (4.1-9.0)	6.6 (4.5-11.7)	0.138
Hematocrit (%)	41.7 ± 2.9	42.7 ± 3.0	0.016
Thrombocytes (10^9/L)	245.4 ± 49.0	246.7 ± 52.5	0.858
**Respiratory medication**			
Respiratory medication use	9 (8.4%)	21 (21,2%)	0.009
Self-reported respiratory medication use	9 (8.7%)	20 (20.8%)	0.015
GP reported respiratory medication use (ATC R03):<br>ICS<br>LABA<br>LAMA	5 (4.7%):<br>4 (3.7%)<br>4 (3.7%)<br>1 (0.9%)	14 (14.1%):<br>12 (12.1%)<br>13 (13.1%)<br>1 (1.0%)	0.019<br>0.025<br>0.014<br>0.956
**Other medication**			
Oral corticosteroids (ATC H02)	0 (0.0%)	3 (3.0%)	N.A.
Oral anti-histaminica (ATC H06)	3 (2.8%)	1 (1.0%)	0.351

SABA = short-acting beta-agonist, LABA = long-acting beta-agonist, LAMA = long-acting muscarinic antagonist, ICS = inhalation corticosteroids, OCS = oral corticosteroids, N.A. = not applicable.
